# Primary pulmonary hyalinizing clear cell carcinoma with vocal-cord squamous cell carcinoma: a case report with systematic review

**DOI:** 10.1186/s13000-023-01376-y

**Published:** 2023-08-08

**Authors:** Zhuo Li, Weihua Li, Liyan Xue

**Affiliations:** 1 Department of Pathology, National Cancer Center/National Clinical Research Center for Cancer/Cancer Hospital, Chinese Academy of Medical Sciences and Peking Union Medical College, No.17, Panjiayuan Nanli, Chaoyang District, Beijing, 100021 China; 2 Center for Cancer Precision Medicine, Cancer Hospital, Chinese Academy of Medical Sciences and Peking Union Medical College, Beijing, 100021 China

**Keywords:** Lung, Head and neck, Hyalinizing clear cell carcinoma, Squamous cell carcinoma, *EWSR1* fusion, Synchronous malignancy

## Abstract

**Background:**

Primary pulmonary hyalinizing clear cell carcinoma (HCCC) is a low-grade salivary gland-type carcinoma. Until now, 23 cases of pulmonary HCCC have been reported.

**Case presentation:**

Here, we present a patient with primary pulmonary HCCC along with vocal-cord squamous cell carcinoma (SCC) revealed by biopsy examination. The patient underwent radiotherapy for vocal-cord SCC, followed by right upper lobectomy and lymph node dissection 10 months later. Histology revealed polygonal cells with eosinophilic or clear cytoplasm in the myxoid matrix together with hyaline degeneration. The tumor involved the whole layer of the segmental bronchus and regionally involved the alveolar tissue along with one intrapulmonary lymph node. Targeted RNA sequencing revealed Ewing Sarcoma Breakpoint Region 1 (*EWSR1*)- activating transcription factor 1 (*ATF1*) fusion. We analyzed the data on pulmonary malignant tumors between 2000 and 2019 in the Surveillance, Epidemiology, and End Results (SEER) database and reviewed all cases of pulmonary HCCC with EWSR1 fusion by searching PubMed. The results showed that head and neck (HN) adenoid cystic carcinoma (ACC) (47.89%) and HNSCC (22.54%) were the most common carcinomas occurring with pulmonary salivary gland-type malignant tumors. Screening of 24 cases of pulmonary HCCC with EWSR1 fusion revealed that five cases demonstrated lymph node metastases and only two had documented tumor recurrences. HCCC is rare and easily misdiagnosed as SCC, but the treatment regimen differs between pulmonary HCCC and SCC.

**Conclusions:**

Hence, pulmonary tumors with clear cells must be diagnosed with caution. Next-generation sequencing (NGS) may be useful for diagnosis, especially in cases with a history of squamous cell carcinoma (SCC).

## Background

Multiple primary malignant neoplasms refer to the appearance of two or multiple malignant neoplasms in the same individual, excluding metastasis from initial primary cancers, and include synchronous and metachronous cancers [1]. Synchronous malignancy of the upper aerodigestive tract is commonly associated with lung cancer [2]. Since lung is a common site of metastasis [3–5], it is difficult to differentiate between a multiple primary tumor and metastasis. A history of primary cancer may play a helpful or interfering role in the diagnosis. Pulmonary salivary gland-type carcinoma is a rare type of pulmonary carcinoma, accounting for less than 1.0% of all pulmonary carcinomas [6]. The differentiation of primary pulmonary salivary gland-type carcinoma from common pulmonary carcinomas such as squamous cell carcinoma (SCC), adenocarcinoma, and intrapulmonary metastases is essential in clinic practice, especially in cases with a history of malignancy [7].

Pulmonary hyalinizing clear cell carcinoma (HCCC) is a salivary gland-type malignancy with good prognosis. Pulmonary HCCC, as a new entity, has been listed in the World Health Organization grading system of thoracic tumors (2021 WHO) [7]. According to the 2021 WHO classification criteria, Ewing Sarcoma Breakpoint Region 1 (*EWSR1*) gene fusion has been identified as a criterion for HCCC. Thus far, 23 cases of pulmonary HCCC with *EWSR1* fusion from 13 articles have been reported. This report describes the first case of pulmonary HCCC with vocal-cord SCC.

## Case presentation

**Fig. 1:**
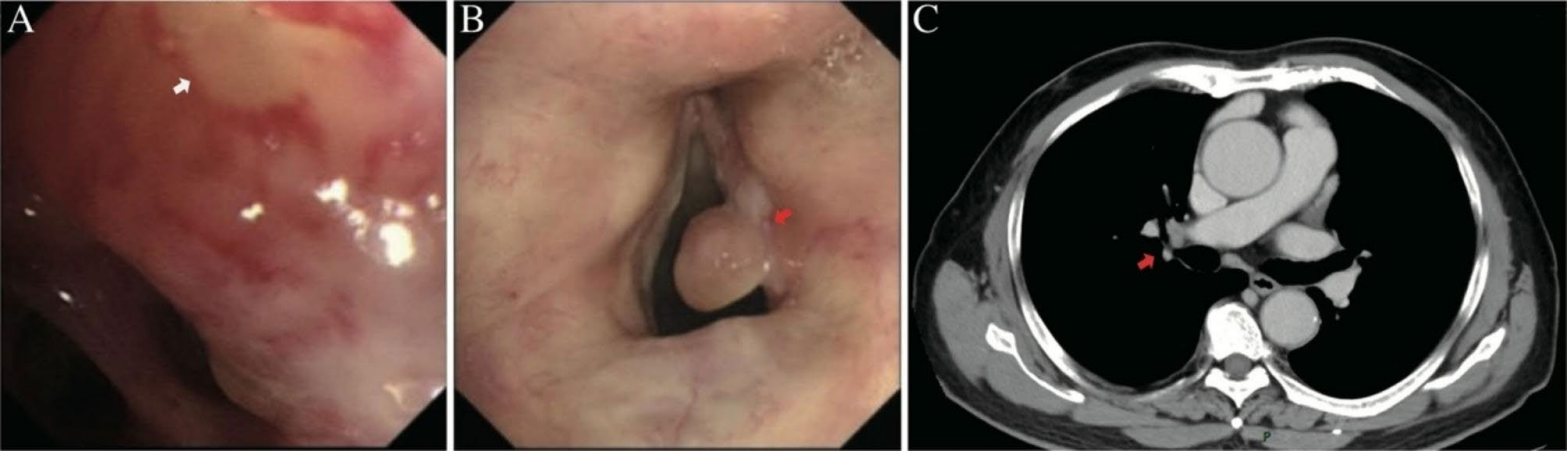
Preoperative representative radiologic and bronchoscopic images from the current case. (**A**) Bronchoscopic findings show a neoplasm arising from the opening of the posterior segmental bronchi of the right upper lobe (arrow). (**B**) Laryngoscope findings showing a polyp on the posterior portion of the right vocal fold with leukoplakia (arrow) (**C**) Computed tomography scan showing a well-demarcated irregular mass in the posterior segmental bronchial root of the posterior apex of the upper right lung (arrow)

An 81-year-old man presented to the Cancer Hospital of the Chinese Academy of Medical Sciences with hoarseness, without a history of smoking, occupational or environmental exposures, or a family history of cancer. Bronchoscopic findings showed a neoplasm arising from the opening of the right upper lobe posterior segmental bronchi (Fig. 1A); and laryngoscopy revealed a polyp on the posterior-portion of the right vocal fold with leukoplakia (Fig. 1B). Computed tomography (CT) revealed a well-demarcated, irregular mass, 2.9 cm in size, in the posterior segmental bronchial root of posterior apex of the upper right lung. The tumor was adjacent to the artery and vein of the right upper lobe (Fig. 1C). Biopsy specimens were pathologically diagnosed as right vocal-cord squamous cell carcinoma with intraepithelial neoplasia and pulmonary HCCC. He underwent radiotherapy 33 times for vocal-cord SCC without any additional therapy for the pulmonary tumor. CT reexamination demonstrated no obvious change 10 months later. He underwent right upper lobectomy and lymph node dissection 10 months later. Histopathologic examination of the resected specimen revealed that the lesion was HCCC with involvement of one lymph node.

### Histopathologic evaluation and immunohistochemistry (IHC)

A fully automated system was used to carry out immunohistochemistry (IHC) on formalin-fixed, paraffin-embedded (FFPE) sections cut at 3 μm. The following antibodies were analyzed on Leica Biosystems: Calponin (EP63, ZSGB-BIO), SMA (1A4, MXB), p53 (MX008, MXB), and p40 (ZR8, MXB). Ki-67 (GM027, genetech), p63 (4A4, Ventana), and Desmin (MX046, MXB) were analyzed on a Discovery Ultra VENTANA systems automated Stainer (Roche), while CK5/6 (MX040, MXB) was analyzed with Dako Autostained Stain System.

### Targeted gene sequencing

Next, 56-gene targeted next-generation sequencing (NGS) was conducted according to the previously described procedure [8]. A QIAamp DNA FFPE Tissue Kit (Qiagen, Düsseldorf, Germany) was used to extract DNA from FFPE tissue. Quantification of DNA was performed using a Qubit 3.0 Fluorometer (Thermo Fisher Scientific, Carlsbad, CA), and quality assessment was performed using a 1% agarose gel electrophoresis. Fragmented genomic DNA was processed to create libraries with barcodes that were used to hybridize with DNA panels. Data from sequenced libraries was analyzed to determine genetic alternations. The closest genes in both directions were defined as the predicted fusion partners when breakpoints were detected in the intergenic regions.

### Patient population

Dataset 22 from the Surveillance, Epidemiology, and End Results (SEER) database was used to analyze the incidence rate of lung malignant neoplasms between 2000 and 2019. International Classification of Diseases for Oncology, 3rd edition (ICD-O-3) was used to identify tumors based on the histological findings. Neoplasms were included according to the following criteria: (1) neoplasms in the head and neck (HN) (C00-C14, C32) and lung (C33, C34) by the ICD-O-3 primary sites code; (2) neoplasms with malignant behavior; (3) primary tumors diagnosed by international criteria; (4) synchronous tumors and metachronous tumors defined as multiple primary tumors diagnosed within 1 year and more than 1 year, respectively.

### Statistical analysis

In this study, multiple primary tumors in the lung and HN were analyzed based on sex, age, sex, primary site, histologic type ICD-O-3, and tumor behavior. The data were compiled by SEER*Stat version 8.4.0.1. and R software (version 4.1.02) was used to generate the boxplot and network.

### Pathologic and IHC findings

**Fig. 2:**
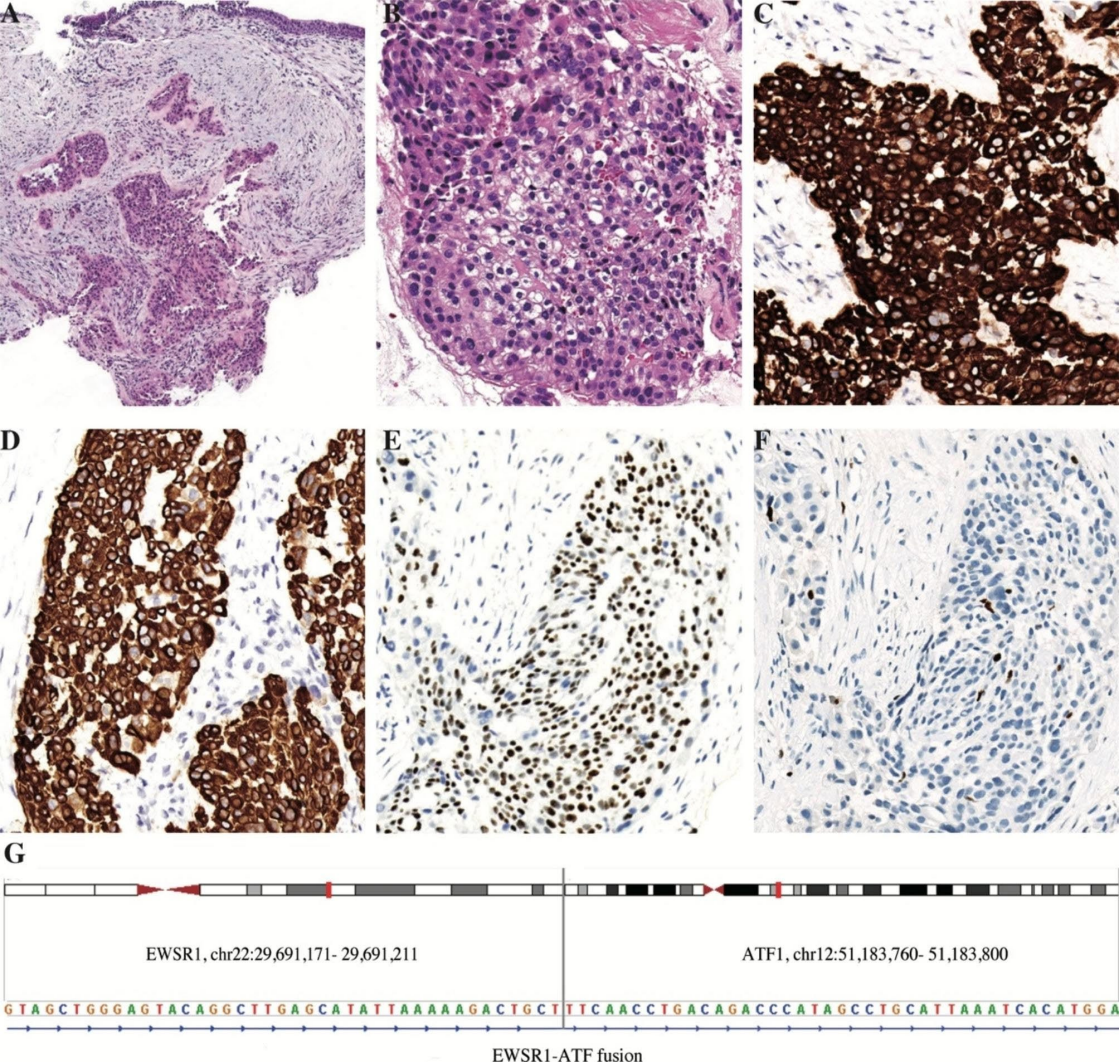
Transbronchial biopsy revealed hyalinizing clear cell carcinoma (HCCC). Tumor cells exhibit infiltrative growth in the myxoid matrix with hyaline degeneration in the bronchial submucosa. (**A**). Tumor cells demonstrate eosinophilic to clear cytoplasm and light atypical round-to-oval nuclei (**B**). Tumor cells express CK7 (**C**), CK5/6 (**D**), and p40 (**E**). Ki-67 index is around 8% (**F**). Targeted next-generation sequencing results show *EWSR1-ATF1* fusion in 14.4% of the tumor cells (**G**)

Biopsy samples obtained by bronchoscopy revealed a neoplasm with infiltrative growth in the bronchial submucosa (Fig. 2A). It was composed of polygonal cells, which were arranged as flaky, adenoid clusters in the myxoid matrix with hyaline degeneration. The polygonal cells had an eosinophilic or clear cytoplasm with light atypical round-to-oval nuclei (Fig. 2B). On IHC, tumor cells demonstrated strongly positive staining for CK7 and CK5/6; mildly positive staining for p40, and negative staining for SMA, Desmin, and Calponin. Ki67 proliferative index was approximately 8% (Fig. 2C-F). According to the morphology of this tumor, NGS was performed. NGS showed *EWSR1-ATF1* fusion in 14.4% of the tumor cells (Fig. 2G). In addition, a frameshift mutation (p. T13fs) in exon 2 of the FANCI gene was observed. Based on the above-mentioned features, the diagnosis of primary pulmonary HCCC was confirmed.

**Fig. 3:**
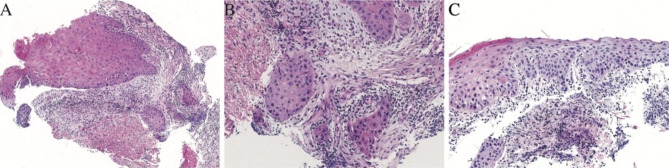
Laryngoscope biopsy reveals well-differentiated squamous cell carcinoma (**A**, **B**) with high grade dysplasia and epidermoid metaplasia (**C**)

Meanwhile, biopsy samples under the laryngoscope yielded a well-differentiated squamous cell carcinoma with high grade dysplasia and epidermoid metaplasia in the right processus vocalis (Fig. 3).

**Fig. 4:**
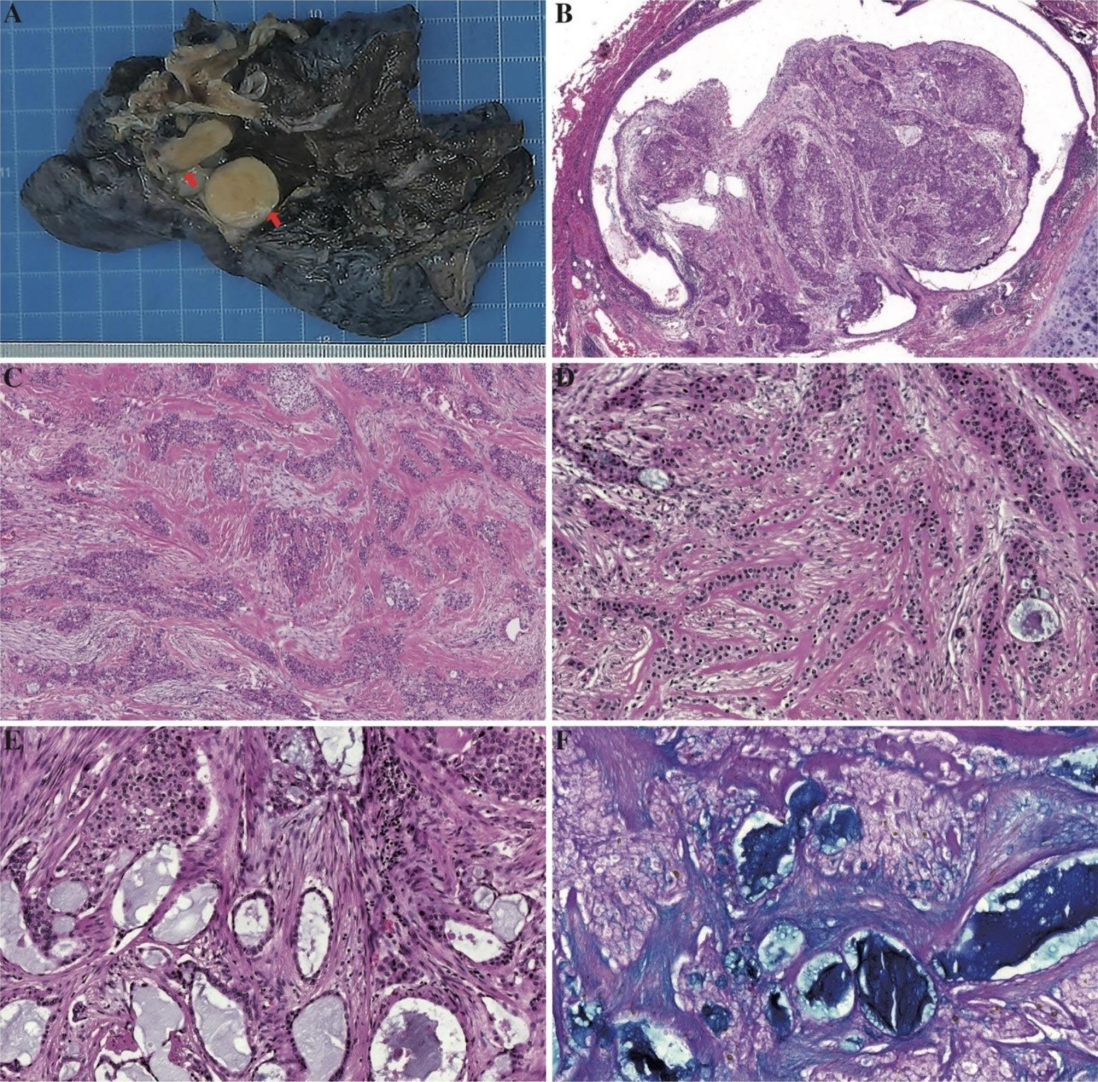
Pathological analysis of the radical resection specimen. On gross examination, the tumor appears yellowish, firm, and solid (**A**). Low power magnification shows prominent endobronchial growth (**B**). Tumor cells are arranged as flaky nests and adenoid clusters in the matrix with hyaline degeneration (**C**). Nuclei are lightly atypical with a small blue nucleolus (**D**). Mucous secretion was observed in the gland-like space (**E**) and confirmed by alcian blue and periodic acid schiff staining (**F**)

A right upper lobectomy with lymphadenectomy was performed. Grossly, a yellowish, firm, solid mass adjacent to the segmental bronchus was identified (Fig. 4A). Hematoxylin and eosin (H&E)-stained sections showed a morphology which was similar to that of bronchial biopsy sections (Fig. 4B-E). Polygonal cells with eosinophilic to clear cytoplasm were arranged as flaky nests or adenoid clusters in the myxoid matrix with hyaline degeneration. Mucous secretion was observed in a gland-like space with no bona fide glandular differentiation. Abundant extracellular mucus with absent mucinous cells was confirmed by alcian blue and periodic acid schiff staining (Fig. 4F). Nuclei were light atypical with a small blue nucleolus and rare nuclear divisions. The neoplasm mainly involved the whole layer of the segmental bronchus and regionally involved the alveolar tissue and one intrapulmonary lymph node. There was evidence of perineural invasion but no pleural involvement or necrosis. The resection margin was negative. In the study period, this patient was free of any local recurrence and required no additional therapy after surgery.

### Multiple primary tumors in the SEER dataset

**Fig. 5:**
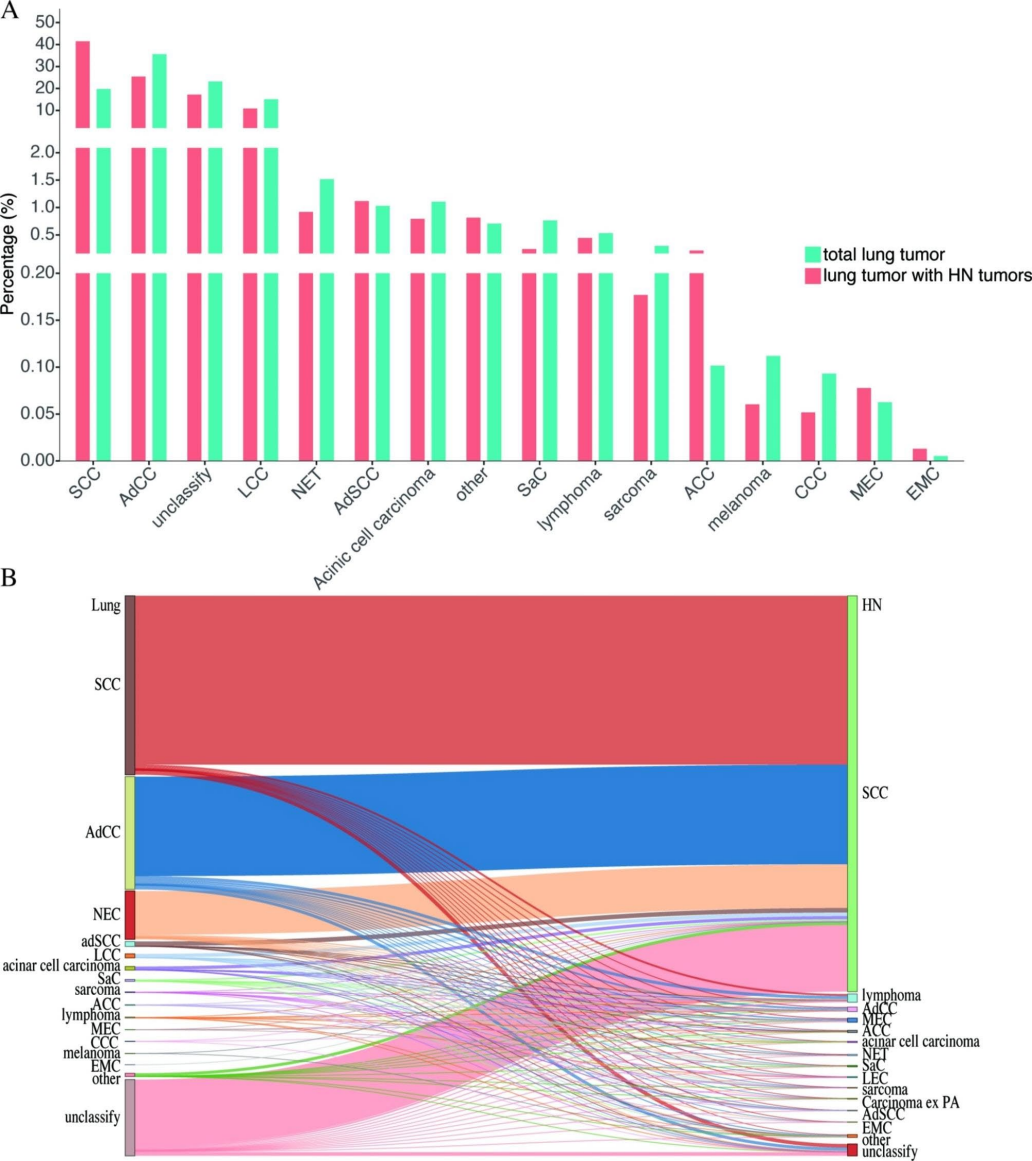
SEER data analysis 2000–2019. Total number of patients with primary pulmonary malignant tumors and patients with metachronous and synchronous primary head and neck malignant tumors (**A**). Sankey diagram shows the relationship of primary pulmonary malignant tumors with metachronous and synchronous head and neck malignant tumors, and the widths of the bands are directly proportional to the number of cases (**B**). HN, head and neck; SCC, squamous cell carcinoma; AdCC, adenocarcinoma; LCC, large cell carcinoma; NET, neuroendocrine tumor; AdSCC, adenosquamous carcinoma; SaC, sarcromatoid carcinoma; ACC, adenoid cystic carcinoma; CCC, clear cell carcinoma; MEC, mucoepidermoid carcinoma; EMC, epithelial myoepithelial carcinoma; LEC lymphoepithelial carcinoma

A total of 1,776,599 primary lung cancers from 1,752,949 patients were selected and analyzed from the SEER dataset. Among them, 21,451 (1.224%) patients had a primary head and neck malignancy tumors before a primary lung malignancy, including 6,049 and 16,689 cases of synchronous and metachronous primary malignant tumors, respectively. Unlike the incidence of total pulmonary malignant tumors, squamous cell carcinoma (SCC) had the highest incidence rate among all pulmonary malignant tumors occurring with HN malignant tumors (Fig. 5). The most frequent patterns were pulmonary SCC with HNSCC and pulmonary adenocarcinoma with HNSCC. Additionally, HN adenoid cystic carcinoma (ACC) (n = 10, 37.04%) and HNSCC (n = 9, 33.33%) were the most common carcinomas occurred with pulmonary salivary gland-type malignant tumors, including ACC, mucoepidermoid carcinoma (MEC), and epithelial myoepithelial carcinoma (EMC).

## Discussion and conclusions

HCCC is a rare salivary gland-type carcinoma with a good prognosis that comprises 1.2% of all primary carcinomas of the salivary gland. According to the 2021 WHO classification criteria, *EWSR1* gene fusion has been identified as one of the criteria for HCCC. Thus far, 23 cases of pulmonary HCCC with *EWSR1* gene fusion from 13 articles have been reported.

The 23 cases of pulmonary HCCC cases with *EWSR1* gene fusion reported in PubMed are summarized in Table 1, along with the present case. Among them, 13 (54.17%) patients were women and 11 (45.83%) were men, with age ranging from 32 to 81 (average, 45.7) years at the time of diagnosis. History of neoplasms was not mentioned in all 23 cases in the literature. Almost all tumors were in the trachea, bronchi, and segmental bronchi (n = 22, 91.67%). Twenty-three patients were treated with resection, only one patient received adjuvant chemoradiation therapy, and one patient received additional adjuvant radiation therapy. Five of the 20 patients who underwent resection and lymph node dissection had lymph node involvement or metastases. Of the 22 patients with available follow-up data, there were only two patients with tumor recurrence. HCCC comprises of polygonal cells arranged as flaky, nested, adenoid clusters in the myxoid matrix with hyaline degeneration. Mucous secretion was observed in gland-like spaces with no bona fide glandular differentiation [8]. Takamatsu et al. found that mucin production and gland-like spaces were slightly higher in frequency in pulmonary HCCC compared with that in salivary HCCC [9].

**Table 1:** Clinicopathologic and molecular features of EWSR1 rearrangement in pulmonary hyalinizing clear cell carcinoma (N = 24)

No.	References	Age	Sex	Site	LN status	Treatment	Outcome	Molecular Testing
1	Doxtader et al. [12]	55	F	trachea	(-)	Resection with radiation therapy	12 months, alive with no recurrence	EWSR1 rearrangement
2	Shah et al. [13]	32	M	bronchi	(-)	Resection	18 months, alive with no recurrence	EWSR1 rearrangement
3	Shah et al. [13]	39	M	bronchi	(-)	Resection	18 months, alive with no recurrence	EWSR1 rearrangement
4	Garcia et al. [14]	38	M	bronchi	NA	Resection	10 months, alive with no recurrence	EWSR1-ATF1 fusion
5	Wang et al. [15]	53	M	bronchi	(+)	Resection	192 months, lung metastasis	EWSR1-ATF1 fusion
6	Jeffus et al. [8]	54	F	bronchi	(-)	Resection	16 months, alive with no recurrence	EWSR1-ATF1 fusion
7	Shahi et al. [16]	55	M	bronchi	(-)	Resection	20 months, alive with no recurrence	EWSR1 rearrangement
8	Takamatsu et al. [9]	52	F	bronchi	NA	Resection	181 months, alive with no recurrence	EWSR1-ATF1 fusion
9	Takamatsu et al. [9]	35	F	bronchi	(-)	Resection	79 months, alive with no recurrence	EWSR1-ATF1 fusion
10	Takamatsu et al. [9]	56	F	bronchi	(+)	Resection	12 months, alive with no recurrence	EWSR1-ATF1 fusion
11	Icard et al. [17]	66	F	trachea	NA	Laser therapy and cryotherapy	NA	EWSR rearrangement
12	Komatsu et al. [18]	56	M	LP	(-)	Resection	6 months, alive with no recurrence	EWSR1-CREM fusion
13	Gubbiotti et al. [10]	46	F	trachea	(-)	Resection and chemoradiation	relapse after 4 years, death after 6 years	EWSR1-ATF1 fusion
14	Wang et al. [19]	57	F	bronchi	(+)	Resection	3 months, alive with no recurrence	EWSR1-ATF1 fusion
15–22	Xue et al. [20]	average: 58 (range: 43–64)	3 M; 5 F	trachea and bronchi	(+):1/8; (-):7/8	Resection	6–45 months, alive with no recurrence (7/7)	EWSR1 rearrangement
23	Chapman et al. [21]	75	F	LP	(-)	Resection	8 months, alive with no recurrence	EWSR1 rearrangement
24	Present case	81	M	bronchi	(+)	Resection	9 months, alive with no recurrence	EWSR1-ATF1 fusion

As far as we know, this is the first reported case of pulmonary HCCC with synchronous vocal-cord SCC. Recent studies have found a significant association between HN carcinoma and pulmonary carcinoma [2]. Data from SEER pulmonary cancer analysis showed that pulmonary SCC was the most common carcinoma occurring with HN malignant tumors. HCCC is rare and easily misdiagnosed as SCC [10]. However, pulmonary HCCC is a low-grade malignancy. The treatment regimen differs between pulmonary HCCC and SCC. Most patients with pulmonary HCCC recover after radical surgery, while a proportion of patients with pulmonary SCC require radical surgery with additional chemoradiotherapy or targeted therapy [11]. Hence, pulmonary tumors with clear cells have to be diagnosed with caution, especially cases with a history of SCC, and NGS may be useful for diagnosis.

In conclusion, the first reported case of pulmonary HCCC with vocal-cord SCC has been described in this report. Our patient had pulmonary HCCC with synchronous vocal-cord SCC, thereby causing significant diagnostic confusion. Multiple tumors with a similar morphology require careful pathological diagnosis.

## Data Availability

The dataset 22 of SEER analyzed during the current study was derived from the SEER database. [https://seer.cancer.gov/statistics/]

## Abbreviations

SCC: Squamous cell carcinoma
HCCC: Hyalinizing clear cell carcinoma
ATF1: Activating transcription factor 1
SEER: Surveillance, Epidemiology, and End Results
HN: Head and neck
ACC: Adenoid cystic carcinoma
NGS: Next-generation sequencing
CT: Computed tomography
FFPE: Formalin-fixed paraffin-embedded
IHC: Immunohistochemistry
